# Solvent Supercritical Fluid Technologies to Extract Bioactive Compounds from Natural Sources: A Review

**DOI:** 10.3390/molecules22071186

**Published:** 2017-07-14

**Authors:** Kooi-Yeong Khaw, Marie-Odile Parat, Paul Nicholas Shaw, James Robert Falconer

**Affiliations:** School of Pharmacy, Pharmacy Australia Centre of Excellence, University of Queensland, Brisbane, QLD 4102, Australia; kooi.khaw@uqconnect.edu.au (K.-Y.K.); m.parat@pharmacy.uq.edu.au (M.-O.P.); n.shaw@uq.edu.au (P.N.S.)

**Keywords:** plants, carbon dioxide, compressed gas, dense gas, pressure, temperature, scale-up, ultrasound, drug discovery

## Abstract

Supercritical fluid technologies offer a propitious method for drug discovery from natural sources. Such methods require relatively short processing times, produce extracts with little or no organic co-solvent, and are able to extract bioactive molecules whilst minimising degradation. Supercritical fluid extraction (SFE) provides a range of benefits, as well as offering routes to overcome some of the limitations that exist with the conventional methods of extraction. Unfortunately, SFE-based methods are not without their own shortcomings; two major ones being: (1) the high establishment cost; and (2) the selective solvent nature of CO_2_, i.e., that CO_2_ only dissolves small non-polar molecules, although this can be viewed as a positive outcome provided bioactive molecules are extracted during solvent-based SFE. This review provides an update of SFE methods for natural products and outlines the main operating parameters for extract recovery. Selected processing considerations are presented regarding supercritical fluids and the development and application of ultrasonic-assisted SFE methods, as well as providing some of the key aspects of SFE scalability.

## 1. Introduction

Natural products have been used as a source of cosmetics, food, and traditional medicines for thousands of years. Plants are the most abundant natural entity on which folklore relies heavily for their pharmacological benefit. For example, the rhizomes of ginger, fruit of *Gingkgo bibola,* and roots of ginseng have all been used extensively as folk medicine with various claimed properties of enhancing cognitive function, preventing nausea, relieving pain, as well as being psycho-stimulants. The notion that a natural product can act as an armamentarium or functional food to prevent or fight an array of diseases such as cancer, neurological disorders, diabetes, bacterial infections, and cardiovascular diseases has helped drive the discovery and extraction methods of modern drugs such as penicillin, taxol, and galantamine. Other examples include, β-carotene, α-tocopherol, α-humerene, carotenoids, unsaturated fatty acids as well as many oils now proven to be beneficial to the health of human beings. In addition, plant-derived products are rich in a variety of secondary metabolites, such as terpenoids, alkaloids, and phenolic compounds [1]. Secondary metabolites are formed by plant biosynthetic reactions and can function as attractants or for defence or signalling purposes [2]. Retrieval of secondary metabolites by extraction is an essential process for drug discovery requiring the recovery of chemical constituents from within a complex matrix of plant material, necessitating the rupture of plant cell walls [3]. There are numerous methods used to extract components of interest, usually initially as a mixture of active and inactive components that are further fractionated to allow the identification of individual molecules. Some examples include basic methanol-based extraction methods or Soxhlet and steam distillation methods; most of these approaches have been used for several decades and can be referred to as traditional methods. Such extraction methods are often simple and practical, unfortunately, they are not without their drawbacks such as being tedious to operate, employing hazardous processing solvents requiring additional steps to remove, and many employing heat thereby resulting in the degradation of heat-labile molecules. Additionally, some of the methods are too selective for only a few types of compounds, although depending on the solvent used, this can mean a large yield of a defined range of compound. Soxhlet extraction, although regarded as the main reference technique to gauge performance of other techniques, is traditionally used for the extraction of lipids [4]. Furthermore, these extraction methods are not only notoriously inefficient, having a poor yield of bioactive extract relative to the high energy input, but are expensive due to the need to dispose of large amounts of organic waste, which in itself can risk environmental issues [5].

The emergence of green chemistry for extraction purposes occurred in the 1990s with the aim of reducing energy consumption and replacing the conventional solvents with less environmentally harmful alternatives [6,7]. Among the greener technologies (that is, greener, by using smaller amounts of organic solvents or by reduced energy consumption through efficient dissolution or reduced processing times) for herbal extraction currently available are ultrasound-assisted extraction, microwave-assisted extraction, supercritical fluid extraction, mechanical pressing, and détente instantanée contrôlée (DIC) [7]. Below is a summary of these technologies (see Table 1). Of these technologies, only supercritical fluid extraction (SFE) offers both reduced processing energy inputs and an alternative solvent approach. Additionally, gaseous solvent recovery on depressurisation from a SCF state (i.e., reversion to a gas), means that phase separation is possible between SCF and the extracted molecules that are in either solid or liquid state. The phase separation can facilitate the collection of pure CO_2_ solvent (gas), such that it can be recirculated into storage, ready for re-use, thus reducing total energy costs (less CO_2_ collection required) thereby reducing energy consumption and increasing the overall sustainability of SCF-based extractions. The technique also avoids the need for solvent waste that is required to be incinerated, an environmentally hazardous and costly process. In should be noted that it is only SFE solvent-based methods that use little or in some cases no organic solvent. Supercritical fluids have been successful as solvents in extraction processes and this can be attributed to their unique tunable physical properties, such as changeable density, liquid/gas-like viscosity and high diffusivity. The application of SFE technologies in the extraction of natural products has been reviewed [8,9,10,11,12,13,14,15,16]. An update of the literature is presented in this review, together with a focus on more environmentally sustainable extraction methods; ultrasonic-assisted SFE methods and the necessary considerations for scale-up.

A supercritical fluid (SCF) of any substance is present where the temperature and pressure are above its critical point (CP), forming a homogenous phase with both liquid-like and gas-like properties—a mesophase. In reality, it can be likened to a compressible liquid or a dense gas, where the liquid-like component does not follow the ideal gas law, which assumes there are no interactions between molecules. Due to its gas-like low viscosity and high diffusivity, a SCF, when used as a solvent, can easily penetrate plant materials with a rapid mass transfer rate. In addition, the density of a SCF can be altered by adjusting the pressure and temperature, hence SCF density is said to be tunable (see Table 2).

In Table 2, it is clear that by simply increasing pressure to four times that of the critical pressure (Pc) of a given SCF, the density can be approximately doubled, thereby nearing that of the density of a liquid, whilst still maintaining the diffusivity and viscosity of that of a gas. This ability to vary viscosity also means that selectivity in extraction of target compounds can be gleaned exclusively by supercritical fluid extraction and within a short period of time (often within one hour). Although a number of solvents such as propane, *n*-butane (or isobutene), methylpropane, Freon (e.g., fluorocarbons), nitrous oxide, dimethyl ether (methoxymethane), and water have been used as SCFs, a number of these solvents have been banned or must meet high health and safety operational standards due to their potentially noxious or hazardous effects on humans, e.g., the critical temperature of water is almost 400 °C [17]. Carbon dioxide (CO_2_) in its supercritical fluid state (scCO_2_) is distinguished as the most commonly used SCF solvent for several reasons: it is readily available, it is a reusable gas, and it has a low critical temperature (Tc) of 31.1 °C and relatively low critical pressure (Pc) of 72.8 bar. In comparison to other SCFs (see Table 3), CO_2_ has a practical or user friendly critical point (CP) as outlined above, as well as having a relatively high CP density of 467.6 kg/m^3^ meaning that its solvent power around the CP is higher than many other SCFs. Although the modification of density is a general property of SCFs, it is scCO_2_ that has a particularly easily tunable density range, e.g., for pure scCO_2_ at 42 °C and 150 bar the density is approximately 766.5 kg/m^3^, while at the same temperature and 400 bar the density increases to approximately 950 kg/m^3^. Further increases in pressure at 750 bar at the same temperature produce a density closer to that of liquid CO_2_, of approximately 1075 kg/m^3^ (the density of liquid CO_2_ is approximately 1256.7 kg/m^3^) [18]. In principle, this means that selectivity in the extraction of certain target compounds could be possible; the selectively could be for just one or two compounds or for a range of compounds, as described in the literature [19,20,21]. In the latter case, a series of high-pressure extraction vessels could be constructed, each with its own temperature and pressure setting, thus isolating any molecule that is soluble in scCO_2_ at those set conditions. It should also be noted that there are different types of processes for the SCF extraction: batch, continuous flow or semi-continuous. The batch process is where the solvent/solute amount is fixed within an extraction vessel and after processing the vessel is depressurised without any further exposure to fresh SCF solvent. In the continuous flow process, the SCF solvent amount is not fixed and fresh SCF is continuously fed into the extraction vessel containing solute. The semi-continuous process arrangement involves a combination of the batch and continuous types of SCF extraction, where the process is switched between the two types with the same sample.

The labelling of carbon dioxide as a green solvent may be confusing or misleading. The carbon dioxide used in this manner is mostly collected as a by-product of chemical manufacturing (e.g., NH_3_ and ethylene oxide) or by trapping methods post-combustion and reforming processes. Following the completion of the SFE process, the CO_2_ reverts to a gas at atmospheric conditions, and, given that stored CO_2_ (bottled or buried) ultimately makes its way back into the atmosphere, the net change in CO_2_ levels is zero. In addition, by using a gas/extract exchange chamber, “clean” CO_2_ (i.e., as a gas—and separated from the extract) can be re-circulated for continued re-use, and this can happen, at least theoretically, over an indefinite time scale. Such a recycling chamber will, however, add to the overall establishment cost and to ongoing running costs and maintenance. Another advantage of CO_2_ SFE methods is the low critical temperature, meaning some of the thermal labile compounds can be retained without damage and extracted. The major drawback of using carbon dioxide as a solvent in its SCF state is that it is only suitable for CO_2_-philic molecules, rendering it useful only for small non-polar molecules, as CO_2_ is intrinsically non-polar. This obstacle can, however, be resolved by the addition of a co-solvent. Furthermore, a SCF can in fact be used as an anti-solvent, where plant material is dissolved in an organic solvent that is then injected into a SCF phase (usually scCO_2_), where the miscible organic solvent phase and CO_2_ interact, reducing the organic solvent phase and the dissolved solutes precipitate, leaving the extract to be examined for bioactivity. The anti-solvent SFE methods have been addressed elsewhere [25,26,27,28,29], and will not be discussed further in this review.

**Figure 1:**
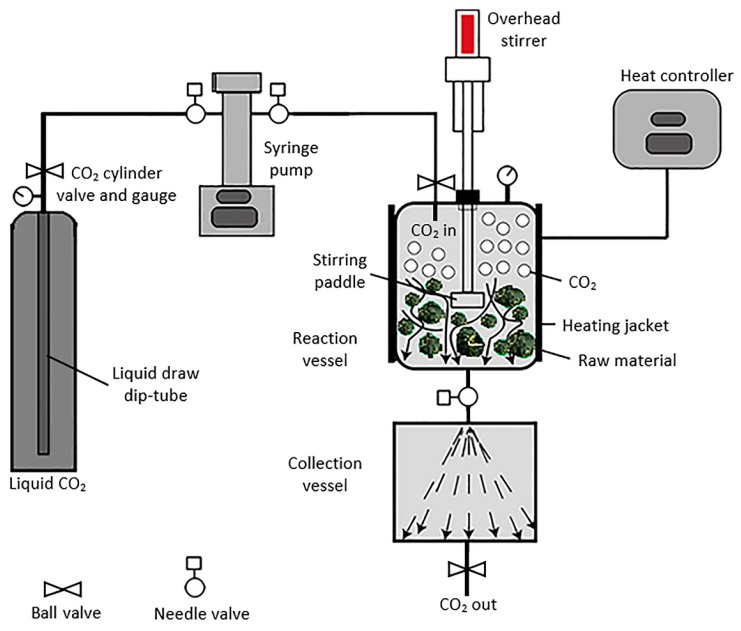
A typical supercritical fluid extraction system.

Supercritical fluid extraction systems (Figure 1) can be divided into laboratory, pilot, or industrial scale. The laboratory scale system aims to produce milligrams to grams of extract by using small volume reactors (50 to 300 mL). Where kilograms of extracts are desired, a system with larger reactor volumes is required with a capacity of several hundred litres [30]. The basic components of a typical CO_2_ supercritical fluid system consist of a CO_2_ pump or compressor, a modifier pump where an organic solvent or water is required, extraction reactor, and fractionation/collection vessel. To achieve the selective extraction of target compound families, sequential separation (using multiple reactors in series) has been introduced as a technique to improve the supercritical fluid extraction [26]. Another method of achieving this is by appropriately varying the pressure, where successive fractionation of classes of compounds can be achieved. Examples include the fractionation of vanilla oleoresins, ginsenosides, and polysaccharides from *Panax ginseng*, or the alkaloids, tocopherols and tocotrienols, and γ-oryzanol from rice bran oil [31,32,33,34]. The yield of the extract is an important outcome measure, and research suggests that the yield of target compounds can be improved by sequential extraction (i.e., multiple extraction vessels connected in series) of natural products using different solvents or even mixtures of solvents at each subsequent stage. Some common solvents used in this type of sequential extraction process have been carbon dioxide, ethanol, and water. For instance, in work published by Monroy et al., the extraction yield (extract weight/raw material weight ×100) obtained from SFE of purple corn cob (*Zea mays* L.) was 0.63% (with CO_2_), 9.7% from the first residue (with ethanol), and 12% from the second residue (with water) with a combined yield of 22.3 ± 1.5% obtained from the sequential reactor method [35].

Extensive research has been carried out to improve the SCF/SFE technique; this research ranges from kinetic modelling [36], sample preparation and pre-treatment by using pressing [37], ultra-sonication [38], microwave irradiation [39], enzyme-assistance [40] or, within the extraction vessel, ultrasonic-assistance [41], and a hydrothermal approach [42]. It should be noted that there are many review publications on the subject of this review, however, most have a large degree of overlap that describes SFE in relation to plant extracts [8,9,10,11,12,13,15] and food [14,16], and there is relative silence in the reviewed literature on the use of sonication in the assistance of SFE-based extractions. The scope of the present review provides a focussed update on research relating to the solvent-based supercritical fluid extraction methods including ultrasonic assisted SFE of plants bearing naturally occurring sources, particularly phytochemicals which have potential nutrient and therapeutic properties. There is a large number of variables that can affect the overall ability and success of extraction by SFE methods, most of which are not the subject of this review; this does not signal their unimportance. Some examples of important SFE variables that are not discussed are, in no particular order; the effects of flow rate, extractor diameter to length ratio (D/L) [43], bed porosity, particle diameter, stirring rate, and a sequential/serial extractor assembly. The scope of this review is focussed on sample preparation, operating parameters, and ultrasonic assisted extraction as well as an assessment of some scale-up challenges associated with SFE technologies and natural products.

## 2. Sample Preparation

Sample preparation is an essential process to help ensure the recovery of bioactive chemical constituents from natural products after cultivation. The choice of drying method, processing raw material size (i.e., ground into course or fine material) as well as sample pre-treatment using methods such as ultrasonication, microwave irradiation, and enzyme assisted degradation of the cellular structure may lead to an improved recovery of bioactive materials.

Raw material pre-treatment chronologically follows plant cultivation; it is therefore important to recognise how different pre-treatment methods such as air flow drying, freeze drying and oven drying may affect the final products recovered. De Aguir et al. studied the effects of different drying methods (freeze drying and oven drying) on capsacinoid content and noted that freeze drying resulted in a higher content of capsaicin and dihydrocapsaicin [44]. It was reasoned that freeze drying provides a less thermally damaging environment to that of high oven temperatures that may otherwise reduce the amounts of recoverable capsaicinoids. Mouahid et al. compared three different drying methods, separately and together; air flow, microwave radiation, and freeze-drying of *Dunaliella salina*. The results showed that air flow drying with microwave radiation produced the highest extraction yields (30.6%) and freeze drying the lowest. It was suggested that air flow drying improved solvent impact on the surface structure and heating via microwave radiation facilitated solvent diffusion as well as improving extraction kinetics [45].

Another important parameter is the particle size of the raw material and this has been claimed to be a key parameter in supercritical fluid extraction processes. Larger raw material particles necessitate longer extraction times, which may consequently influence yield. In contrast, a smaller starting material particle size is associated with a larger surface area and diminished intra-particle diffusion resistance, as the diffusion path is reduced, thereby accelerating the extraction process and efficiency [46]. For example, Özkal and Yener showed that, by decreasing the particle size, the yield increased by 24% in the SFE of flaxseed oil [47]. In addition, the optimum particle sizes (diameter) for the extraction of the *Garcinia mangostana* were a mean of 0.9 mm for total phenolic compounds and 0.6 mm for α-mangostin [42]. However, further reductions in particle size have been noted to reduce extract yields, possibly due to agglomeration, a function of strong cohesive forces between particles, causing CO_2_ flows only through micro-channels with reduced surface area [48]. There is also the issue of entrainment, which can be problematic when using too much raw material and/or raw material that has been too finely ground. One method to help prevent entrainment has been to utilise a mesh canvas over the extractor inlets, as was reported by Barroso and colleagues. In this study, papaya seeds were crushed using a pestle and 6.5 g fed into an extractor with a 42 mL capacity [49]. Experimental yields (extract mass/free solute mass ×100) ranged from 0.44 to 2.56% as a function of temperature and pressure.

The use of conventional extraction methods assisted with either ultrasound, enzyme pre-treatment, or the use of microwave radiation have become popular SFE-adjuvant options. For example, microwave pre-treatment at 100 W for 30 s of *Moringa oleifera* seeds increased the yield of the oil by 11% with the final oil yield being 34.05% [39]. Recently, enzymes have also been used to assist supercritical fluid extraction processes [40,50,51]. Cellulases, pectinases, and hemicellulases are common enzymes used to degrade the structural integrity of the plant cell wall through hydrolysis. For example, the use of α-amylase in pre-treatment of black pepper prior to supercritical fluid extraction increased the yield of extract and piperine-rich extract by 53% and 46%, respectively [51]. Another example involved the use of recombinant enzymes in the pre-treatment phase of pomegranate peel, followed by SFE [50]. The result showed that a mixture of enzymes (cellulose, pectinase and protease) in the ratio of 2:1:1 amplified the extraction yield and the total phenolics content by two-fold compared to that of the control (solely supercritical fluid extraction). Another example was the extraction of lycopene from tomato skin, where a combination of cellulase with a carbohydrase multi-enzyme complex selectively increased the yield [40]. Therefore, sample pre-processing can amplify the yield of extraction methods and is worthy of continuing research [52,53].

## 3. Operating Parameters

### 3.1. Effect of Temperature and Pressure

Pressure and temperature are the key parameters controlling the SCF extraction process; an increase in pressure results in an increase in fluid density and enhanced solubility of the solute [8]. Belayneh et al., who performed SFE studies on seed of *Camelina sativa*, reported high oil yields (31.6%) at high pressure with operating conditions of 70 °C, 450 bar and 510 min [54]. Another example is the extraction of oil from seed of *Gynostemma pentaphyllum*; Wang et al. demonstrated that increasing pressure from 150 to 300 bar marked an increase of 53% in the yield of oil [55]. Nevertheless, increasing pressure to a certain point may reduce the diffusivity of the SCF solvent and result in a reduced contact with pores in the raw material, thereby potentially reducing solute dissolution [53]. In addition, in some cases, an increase in pressure may cause the solid matrix to compact and the void fraction leads to unfavourable extraction outcomes [45,56,57]. For instance, Shao et al. reported a decrease in oil recoveries by increasing pressure above 306 bar [58]. However, one of advantages of SFE is the ability to selectively extract bioactive phytochemicals of interest with note, the extraction pressure for phenolic (350 bar) and anthocyanin (220 bar) from the pulp of *Euterpe oleracea* [59]. Other examples, as listed in Table 4 where there appear to be optimal extraction pressures, include, α-tocopherol from *Persea americana*, thymol from *Lippie sidoides*, and eugenol from *Ocimum sanctum* L. [60,61,62].

It is essential to understand the relationships between pressure and temperature for a SCF, in which both factors directly impact on its physical properties; that is density, viscosity, and diffusivity. Temperature can exert an inconsistent “solvent” effect, where a higher temperature confers more “energy” to a fixed wall system, increasing diffusivity, and increasing the apparent volume, and density reduces, along with the “solvent” power of the SCF. On the other hand, decreasing the temperature decreases the vapour pressure of the solutes, and the density rises, along with the SCF solvation of the solutes. This results in a phenomenon called the “crossover effect” where high temperatures can produce low yields, while low temperatures can produce a high extract yield. Ghosh et al. showed that the recovery of eugenol from *Ocimum sanctum* was decreased significantly by amplifying temperature at lower pressure and vice versa, and the upper cross-over pressure was noted at 200 bar [60]. Other examples where extract yield and SCF density effects have been noted are for γ-oryzanol with a cross-over effect at 300 bar [97], 350–375 bar for avocado oil [61], as well as 295 bar for coconut oil [98].

It should be noted that our understandings about SCFs and the crossover region are related to single solutes, and not to complex mixtures of solutes such as extracts. For example, in some studies, a higher temperature resulted in higher yields, contrary to crossing-over of density effects. Nejad-Sadeghi et al. reported that the yield of essential oil was directly proportional to the temperature and speculated as to the dominant effect of diffusion over density [48]. The authors reasoned that increases in kinetic energy from increasing temperature were directly proportional to the rate of diffusion of CO_2_ within the raw plant material. This was in contrast to work done by Ben-Rahal et al., who noted that the recovery of oil from milk thistle seeds decreased as a result of increases in the temperature and this arose from the effects of solvent density [84]. It is clear that each plant and its extracts may have variable yields with changing SCF conditions, arising from SCF conditions that will dissolve different amounts of extract solutes, and thus is likely to be distinctive for each plant species investigated.

### 3.2. Effect of Organic Modifier

Supercritical carbon dioxide is intrinsically non-polar and it is an excellent solvent for extraction of non-polar compounds and some low molecular weight, volatile, polar compounds. However, it is less effective in the extraction of polar phytochemicals embedded in the cell wall. Nevertheless, the addition of small amounts of organic co-solvent or solvent modifier in the SFE process can enhance the solvation power of scCO_2_ and improve the recovery of bioactive compounds [30]. In addition, the inclusion of a modifier may counter the activation energy of the analyte desorption and intensify the transportation of the analyte to the fluid [99,100]. The most commonly used organic solvents in this regard are ethanol and methanol. One study that investigated the extraction of polyphenols used 15% *v*/*v* water: scCO_2_ [94], followed by a 15% *v*/*v* ethanol:scCO_2_ extraction step; this method gave a higher recovery of up to 7.3%, compared to that of the method without the additional ethanol step. The yields were 63.4g/kg of extract and 68.0 g/kg of extract, respectively. A proportion of co-solvent has proved essential in recovering certain highly polar compounds; e.g., the extraction of proanthocyanidins from grapes using 15% *v*/*v* of ethanol-water as co-solvent [94]. Gañán et al. demonstrated the extraction of benzyl isoquinoline alkaloids namely chelidonine, chelerythrine, sanquinarine and berberine from root of *Chelidonium majus* by SFE [31]. The optimum extraction conditions for such alkaloids were 55 °C, 120 bar and basified ethanol as co-solvent. Remaining to be studied are the effects of solvent mixtures under high pressure with varying temperatures. As alluded to in the previous section, the effects of temperature and pressure on the dissolution of solutes in any given plant extraction system are not necessarily readily predicted, and the addition of co-solvents further complicates issues; interactions may then occur not only between CO_2_ and CO_2_ and solutes, but also between CO_2_ and co-solvent. It would be likely that in some cases the use of a co-solvent may reduce the targeted extraction of bioactives, as CO_2_/co-solvent interactions reduce the availability of CO_2_ as a solvent of certain molecules, even if the overall yield is increased.

### 3.3. Effect of Water in SFE

Water content is one of the key aspects in determining the quality of the output of SFE processes. Water is considered to be soluble at approximately 0.3% *v*/*v* [8] in supercritical CO_2_. The presence of water, however, may either assist in or be an impediment to the diffusion of supercritical carbon dioxide; what is necessary for effective extractions depends on the type of compounds targeted [8]. For example, the moisture content of paprika is reported to be as high as 85% [101] and SFE extractions result in extremely low yields. Balasubramanian et al. showed that biomass moisture content as high as 5% had no effect on lipid extraction efficiency from the marine microalgae, *Nannochloropsis* sp. [102]. Higher water content in pre-treated raw materials may also be acceptable, as Crampon et al. reported complete recovery of neutral lipids from *Nannochloropsis oculata* with water content up to 20% of weight [103]. In another study, Ivanovic et al. verified the effects of moisture content on the extraction of essential oils from the flowers of *Helichrysum italicum* and they observed that pre-soaking of the samples in water (moisture content: 28.4% *w*/*w*) led to an increase in the extraction yield of 40% and reduced CO_2_ consumption (decreased by 25%) with the operating parameters of pressure at 100 bar; temperature at 40 °C; CO_2_ density 630 kg/m^3^ [104].

## 4. Ultrasonic-Assisted Supercritical Fluid Extraction

Ultrasonic waves are able to generate mechanical deformation in solid, liquid and gaseous media and are characterised by a frequency range from 20 kHz to 10 MHz [7]. It is considered to be an environmentally sustainable addition to existing extraction methods by providing cleaner extracts while using less solvent and shorter extraction times when compared to conventional methods. Ultrasound techniques have been employed in SFE as a sample pre-treatment step and also during the SFE process, see Table 5. As an example, ultrasonic pre-treatment of hemp seed at 200 W for 10 min significantly improved the yield of the oil by 3.3% [105]. However, longer durations of extraction at 20 and 40 min proved undesirable as they led to sample degradation by oxidation, by polymerisation, as well as by non-enzymatic browning reactions [105]. In contrast, Said et al. reported that the pre-treatment of ginger by ultrasound over a 60 min period increased the yield of oleoresin by approximately 110% as compared to methanol extraction without ultrasound [106].

Waterbath-based systems are a means of using ultrasound during SFE (placing the extraction vessel without heating wires directly into a sonicating bath), while the use of an ultrasonic probe would be used as a pre-treatment process. There are SFE systems that are capable of housing a sonotrode, however, there does not appear to be literature available regarding its use in SFE. Both ultrasound processes, either used as a pre-treatment or by using a waterbath, were reported to enhance the extraction rate and yield through mechanical stirring and the main driving force may be attributed to cavitation phenomena [107]. These systems have been applied to assist in the extraction of docosahexaenoic acid from microalgae and oil [108,109]. As one of the aims for employing ultrasonic systems with SFE to improve the yield of bioactive phytochemicals, Kawamura et al. demonstrated that the yield of luteolin and apigenin from leaves of *Perilla frutescens* increased by 53% and 144%, respectively, using ultrasound exposure for 125s. In addition, Santos et al. performed extractions of capsaicinoids from the fruit of *Capsicum frutescens* and found notable increases in these compounds by up to 77% when an ultrasound probe was used. In another study, the yield of oleonolic and ursolic acid from *Hedyotis diffusa* increased by 11–14% following treatment with ultrasound generated from a sonicating waterbath. From these results, it may be deduced that the use of an ultrasonic probe represents a more promising approach than that of waterbath-based ultrasound to amplify extraction yields. It may be reasoned that close contact of the probe with the matrix results in a more direct delivery of ultrasound into the media, leading to greater rupturing of cell walls and more solvent penetration and mass transfer. A major deficiency of employing an ultrasound probe is the rapid increase in temperature, fostering the potential degradation of thermally labile compounds. Therefore, a short period only of ultrasonic treatment is recommended in order to preserve the labile chemical components. More research is required to determine the value of ultrasonic-assisted SFE, taking into account time, cost, and whether the results are reproducible at a larger scale.

## 5. Scale Up

The evolution of extraction methods from the conventional to the more advanced, represented by such as SFE-based technologies, highlights the scientific need for ever-improving extraction technologies in the pharmaceutical, food and life sciences. An important aspect in the application of contemporary extraction methods is the ability of typically used equipment to be increased in size without any significant effect on the results produced, e.g., from laboratory scale (50 to 2000 mL extraction vessel) to pilot scale (5 to 10 L extraction vessel) extractions. From the literature, a range of criteria have been reported [12,115,116] for scaling up SFE-based technologies, although there appears to be only minimal agreement on what the key criteria are, indicating that further research on the matter is required. One theme that is consistent with any form of scale-up criteria (and is not only applicable to SFE) is that the results of the optimised laboratory scale mimic those of the production scale [115]. In addition, economic feasibility and the impacts of commercial operations feed into scale-up considerations; there are various strategies used to provide an expected outcome, including mathematical formulae to provide cost estimates of manufacturing. The terms of such formulae may include labour costs ($/year), utility cost ($/year), waste management cost ($/year), raw material cost ($/year), and initial investment ($/year) [116,117]. The cost estimations are likely to vary widely due to the different specifications for each method, and thus different equipment requirements would likely have different costs. However, one way to estimate costs, which has been proposed by Rocha-Uribe et al. [118] is to use data from the Chemical Engineering Plant Cost Index (CEPCI). Using 2013 data, the cost of a production scale SFE unit was estimated at US$ 2,540,843.00 [119]. Another possible source of variation is in the labour expenditure estimates, where plant operations in Asia, Europe, North America, and South America will have different manpower costs, as well as having a range of cost differences for specific operational factors such as electrical power and waste management.

In terms of some of the technical criteria that are exclusive to SFE and scale-up; there appear to be a number of common ones that have been based on upscaling studies of promising SFE research. For example, Prado et al. (2011), while studying the SFE process for clove and sugarcane residue, proposed that maintaining a constant solvent mass (S) to feed mass (F) ratio, would generate minimal interference with the extraction yield [117]. While a review by de Melo et al. (2014) on SFE of vegetable matrices, outlined the following key criteria [12]. The first, similar to the ratio described above, is to fix the mass of SCF solvent per mass of bed raw material, especially when solubility is an underlying process limitation. The second criterion is that when diffusion limits the process, the maintenance of SCF flow rate to mass of bed raw material is important. Where both solubility and diffusivity are both considered to be important limiting processes, then both factors ideally should be controlled as closely as possible i.e., kept constant [12]. When SCF parameters such as temperature and pressure must be held unchanged, the use of dimensionless ratios can be used such as Reynolds number (a value in fluid mechanics representing laminar vs. turbulent flow properties, thus inertial vs. viscous forces, respectively). Finally, as a separate criterion to those listed previously, consideration of geometric similarities has been proposed [12]. In this approach, the ratio of vessel bed length (height) vs. vessel bed diameter could be useful for scale-up purposes. It is also important to note that, despite having controlled as many criteria as might seem reasonable, larger scale results may not necessarily correspond to those achieved at smaller scales. For example, entrainment (or biomass aggregation and blockage of piping) may be less relevant at laboratory scales but become a significant issue at pilot or production scale [12]. Therefore, some architectural and engineering design factors may become more relevant, where a set of stainless meshes or activated charcoal barriers and stacked chambers may be introduced to reduce the chance of any large-scale entrainment process.

Another way to undertake scale up processes, according to Pronyk and Mazza, is to consider that the extraction process occurs in two different stages: the first being external, followed by internal mass transfer [120]. The first external mass transfer process is due to oil or solute that is freely present or diffused to the surface of the raw material; the second internal mass transfer is due to the extraction that is controlled by the movement of the solvent within the material. In addition, the SCF dissolution of solutes within the solid matrix is a rate-limiting step, which is particularly problematic for plant materials, due to the complex nature of their matrix [121], as well as the phase behaviour effects of SFE itself [122]. Some processing parameters that influence the scale-up process include the solubility of the compound(s) of interest, flow rate, raw material particle size (and distribution) as well as the design of the extraction vessels such as bed geometry [120]. Although many factors influence the effectiveness of scale up process, employing mathematical models may offer a risk-reduction approach to scaling up by allowing the operator to define and control the limiting factors governing the SFE process [123].

As alluded to above, bed geometry is one of the factors that may affect SFE outcomes and is also related to the cost of a reactor vessel, this being primarily a function of its diameter. Paula et al. studied the influence of bed geometry and cost of SFE by using different sized fixed bed reactors with height to diameter ratios (H/D) ranging from 1.86 to 5.2 for the scale up extraction of *Baccharis dracunculifolia* leaves. The results showed similar kinetic behaviours for all reactor types suggesting that solvent flux was kept constant [119]. The chemical constituents recovered from the leaves were the same and in similar amounts (*w*/*w* basis) and included caffeic acid, *p*-coumaric acid, *trans*-cinnamic acid, artepillin C, and kaempferide. In addition, it was found that the optimal manufacturing cost was estimated at US$ 171.22/kg at 80 min, suggesting that industrial scale operation and outputs are possible for SFE of *B. dracunculifolia* leaves.

In another study, de Melo et al. conducted SFE experiments to recover triterpenic acids from the bark of *Eucalytus globulus* by using different sized extraction vessels (0.5, 5 and 80 L) [124]. Experiments were conducted by employing laboratory to pilot scale vessels (using equipment with extraction vessel volumes of <10 L) with the aid of modelling to define the mass transfer mechanisms; a key scale up criterion (as noted above). The extraction yield and triterpenic acid concentrations were similar in scale up from laboratory to pilot scale, facilitated by maintaining CO_2_ flow rate and biomass weight ratios during the SFE process [124]. Other studies have performed experimental and simulation models in the extraction of leaves of *Mangifera indica* with three different extraction methods; namely SFE, pressurized liquid extraction (PLE), and enhanced solvent extraction (ESE) [125]. By employing a mathematical model (Sovová’s cellular model) and by using a combination of similarity theory and dynamic parameters, the results showed that ESE and PLE gave 4.6-fold higher recoveries than that of the SFE method. In addition, it was noted that a constant solvent mass-to-feed ratio and bed geometry proved useful in the scale up process. It is noteworthy that SFE processes are governed by diffusion, whereas PLE and ESE processes are largely controlled by solubility (and density), although the involvement of internal diffusion mechanisms in the recovery process is probably still present.

## 6. Outlook of the Field

SFE solvent methods have been used extensively to extract compounds of interest from a wide variety of natural sources, as outlined in Table 4. Although the popularity and research investment in SFE methods predates the 2000 s, there is still many gaps in the literature, such as drug discovery from indigenous plants across the globe, all of which could be investigated for their biological activities against, to name a few; cancers, cardiovascular disease, inflammatory disease, bacteria, and Alzheimer’s disease. There is also the requirement for continued research into improved SFE methods in the food supplement and nutraceutical sector. This is an important recent research line that can be developed further using SFE biotechnology and can contribute to preventative medicine and healthier living.

One other drawback that should be noted is the technical application of SFE. While SCFs are a “tunable solvent” and this is an incredible parameter to be able to utilise, this can in fact be their Achilles heel. The ideal extraction of solute from different natural sources will change the phase boundaries, mainly vapour/liquid equilibrium (VLE) and critical point, thus variable solvent/solute solubilisation and diffusion. Such changes could be vastly different with even very little change in temperature or pressure, so making a hypothesis and experimental design and method development a relatively challenging task. Although, phase behaviour can be complex, even for binary systems, simple *P-T* phase diagrams and *P-x* diagrams can help with making progress. Although, large gaps exist in the literature, there is still enormous amounts of information on the matter, some starting points and reviews are offered here [62,126,127,128,129,130].

Although the cost of SCF technology has been diminishing, this decrease is not significant and the high cost of equipment remains a major drawback in implementing SCF applications, including SFE. In addition to the high cost of compressors, the high pressures required in the reactor/extraction vessels also need to be maintained across transfer tubing, valves, connection points, etc. and all must meet appropriate safety codes, which adds to the initial setup costs. The SFE advantages, as listed in Table 1, however, outweigh the drawbacks, and there are industrial success stories such as the decaffeination of tea leaves by Evonik Industries in Germany, illustrating the fact that SFE solvent technologies can operate with a viable business model. To highlight just a few of the overarching advantages in this section, the actual application of extractions by SCF are simple, non- detrimental to proteins in their structure and function, including enzymes, the processing itself is inexpensive and when using carbon dioxide for SFE, the SCF solvent phase is non-flammable and non-toxic to the material exposed to it, as well as being easily removed from any final extract. Further to this, is the potential to “select” important solutes by SFE, which cannot be overstated. For most SFE methods overall yield of extract would often be greatly reduced, the increased extraction of desired solutes by SFE renders this method with one of the highest efficiently ratings, thus a superior extraction method compared to that of conventional methods. For solutes that require co-solvents to be extracted by a solvent SCF driven method, then polar solvents can be added to CO_2_ in quantities up to 5% *v/v*. This also renders SFE methods a green/er alternative to conventional methods, as either no organic solvent is necessary or only small amounts are used that can be “spray dried” to be effectively “eliminated” from the extract, resulting in negligible amounts of organic solvent residue in an extract. SCFs will continue to play a significant role in the extraction of natural sources and their tunable physical properties offer a unique variable, as highlighted in the review by Farrán et al. [131]. Furthermore, current non-SCF extraction technologies are heavily reliant on organic solvents, producing waste product that must be incinerated by burning of fossil fuels which is an unsustainable practice in the longer run. Academic research must continue to increase in this field so that SCF methods are well placed to fill the gap once industry and regulators decide to switch to alternative technologies. In the next decade, SCFs and their applications are likely to hold a critical part in the development of sustainable technologies.

## 7. Conclusions

Over the last 20 years, the use of supercritical fluid extraction research has increased in the field of natural products. SFE methods have been used to harvest a large range of extracts from oils, oleoresin, groups of bioactive compounds (alkaloids, terpenes, and phenolic) as well as single compounds (α-humulene, lycopene and α-tocopherol). Solvent SCF offers a more selective and environmentally sustainable alternative to traditional methods in natural products extraction; solvent SCF-based extraction methods do not require organic solvents. In addition, solvent SFE may offer improved selectively of bioactive extraction, while the use of ultrasonic-assisted SFE may offer yet greater improvements in the yield of bioactive compounds due to a high mass transfer rate. Collectively, SCF-based extraction methods are a technology with large potential and thus merit further investigation.

## Figures and Tables

**Table 1:** The advantages and disadvantages of greener extraction methods.

Method	Advantages	Disadvantages
Microwave-assisted extraction (used with traditional methods)	rapid extraction; small amount of solvent; relatively low additional costs	use of high pressure and temperature; limited amount of sample; non-selective (large number of compounds extracted)
Supercritical fluid extraction (SFE) methods	rapid extraction; small amount of organic solvent or no solvent; no solvent residue; preserves thermally labile compounds; tunable solvent (SCF) density; selective extraction (small number of compounds extracted); inexpensive to operate/run	high setup cost; technical knowledge of SCF properties required (e.g., phase behaviour, cross-over region)
Mechanical extraction	mainly for extraction of oil and juice; does not require external heat and solvent	limited application and non-selective
Ultrasound-assisted extraction (used with traditional methods)	rapid extraction; small amount of solvent; relatively low additional cost	non-selective
DIC extraction—détente instantanée controlee (steam driven with rapid depressurisation)	improved extraction yield; rapid extraction	high cost; high energy consumption; high temperature; preferably used for sample pre-treatment process

**Table 2:** The relative properties of liquid, gas and SCF phases.

Phase	Physical Property		
	Density × 10^2^ (kg·m^−3^)	Diffusivity × 10^−3^ (cm^2^·s^−1^)	Viscosity × 10^−4^ (kg·m·s^−1^)
Liquid	6–16	<0.005	2–30
SCF			
Pc, Tc	2–5	0.7	0.1–0.3
4Pc, Tc	4–9	0.2	0.3–0.9
Gas	0.006–0.02	0.1–0.4	0.1–0.3

Key: Pc = critical pressure, Tc = critical temperature.

**Table 3:** Apparent physical properties for various supercritical fluids [22,23,24].

SCF	Molecular Weight	Critical Temperature	Critical Pressure	Density at CP ^†^	Notes
	g·mol^−1^	°C	bar (psi) *	kg·m^−3^	
Air	n/a	−140.6	37.7 (546.8)	319.9	Green technology fluids and relatively higher CP densities
Ammonia (NH_3_)	17.03	132.2	113.3 (1643.2)	225	
Nitrogen (N_2_)	28.01	−147	34 (493.1)	313.3	
Water (H_2_O)	18.02	373.9	220.6 (3166)	322	
Carbon dioxide (CO_2_)	44.01	30.9	73.7 (1056)	467.6	Greener ** technology and high CP density
Chlorotrifluoromethane (CCIF_3_)	104.5	28.8	38.8 (563.3)	582.9	Higher CP densities but environmentally hazardous
Dichlorofluoromethane (CHCl_2_F)	102.9	178.3	51.8 (751.3)	526.1	
Octafluoropropane (C_3_F_8_)	188	71.9	26.8 (388.7)	629	
Acetone (C_3_H_6_O)	58.08	235.1	46.4 (672.9)	278	Lower CP densities and environmentally hazardous
Benzene (C_6_H_6_)	78.11	289	49 (710.7)	30.9	
Dimethyl Ether (CH_3_)_2_O	46.1	127.1	53.4 (774.5)	277	
Ethane (C_2_H_6_)	30.07	32.2	48.7 (697.6)	206.2	
Ethanol (C_2_H_5_OH)	46.07	240.9	60.6 (878.9)	276	
Ethylene (C_2_H_4_)	28.05	9.2	50.4 (720.9)	214.2	
Methane (CH_4_)	16.04	−82.6	45.9 (658.5)	162.7	
Methanol (CH_3_OH)	32.04	239.4	81 (1157.4)	275.5	
n-Propane (C_3_H_8_)	44.1	96.7	42.5 (761.4)	220.5	
Propylene (C_3_H_6_)	42.08	91.9	45.5 (658.5)	230.1	

Key: * Pressure conversions: 1 MPa = 10 atm or bar = 145 psi = 2059 kg·cm^−2^. Pressure will be presented in bar hereafter. ** Greener = being extracted from the atmosphere for SCF processing and where scCO_2_ acts as a solvent it can replace organic solvents. ^†^ CP = critical point.

**Table 4:** Solvent supercritical fluid extraction of natural products.

Natural Source (Scientific Name)	SFE Conditions					Major Findings about the Extraction/Extract				
	Temperature (°C)	Pressure (bar)	Flowrate (g/min)	Processing Time (min)	Dimension Extractor (D or ID × L)	Solvent/Solid Ratio (gCO_2_/g) *	Identified Molecule/s	Amount of Active	Reference	Remark/s
Industrial examples	35–38	248	-	-	7003 L	-	caffeine	-	[63]	Decaffeination of tea leaves by SFE, Evonik Industries AG (Essen Germany)
Wide variety of (Tea leaves) *Theaceae*										
Industrial waste: Tomato skins and seeds	60	300	0.16, 0.27, and 0.41	3–8 h	5 mL ID = 7.9 mm	220	*E*-lycopene	86% recovery of *E*-lycopene	[64]	Lab-bench scale and the result was from the CO_2_ only method
Agricultural by-product	35	140	0.339	-	6 mL	-	lignin derived bioactive compounds e.g., tricin and catechins	-	[65]	SCF used as the separation of fractions from ionic liquids. Relatively poor extractions yields of flavonoids was explained by the higher polarity of catechins than vanillin-like compounds
*Wheat Straw*										
Vinegar	50	350	0.42	120	-	-	44 aroma compounds e.g., acetic acid, benzaldehyde, ethyl acetate	-	[66]	SFE is used to recover highly prized aromas from a Chinese vinegar
*Zhenjiang*										
Orange peel	35	131	33.33	-	-	-	Wide range of oxygenated compounds	-	[67]	Used as a second step to recover flavor compounds from a silica gel bed
*Citrus sinensis* (Orange oil)										
Passion fruit	50–60	170–260	20.64	-	54.37 mL (30.3 mm × 75.4 mm)		tocopherols, unsaturated fatty acid and carotenoids	-	[68]	Sequential SFE method marked an increase in retrieval of bioactives compared to single step SFE method
*Passiflora edulis* (Passion fruit bagasse)										
Grape marc	40	200–500	0.41–32.78	-	Used a series of extractors (0.1, 0.2, and 0.5 L)		UFAs and vitamin E (tocopherols and tocotrienols)	-	[43]	Study investigated extraction kinetics using SFE and grape marc
Grape seed oil										
Olive husk	40–60	205–350	15L/min	180	0.94 L (40 mm × 75 mm)		tocopherols, carotenoids and chlorophylls	-	[69]	Two-to-four times increase in recovery of bioactive compounds compared to conventional method
*Oleaceae*										
Dairy	35 and 40	200 and 350	-	-	-	-	non-polar lipids; triacylglycerides and FFAs	-	[70]	Reduction of fat content by 51% for cheddar and 55% for Parmesan
*Cheddar and Parmesan cheeses*										
Plant example	40–50	300–400	0.39	-	-	-	luteolin, carajurin, 3-desoxyanthocyanidin	19 mg/g leaf	[56]	Authors explored selectivity of SFE in extraction of phenolic compounds
*Arrabidaea chica*										
*Benincasa hispida* (Winter melon)	46	244	10	97	500 mL	-	linoleic acid	176 mg extract/g dried sample	[71]	Antioxidant activity of SFE extract was higher than in extracts derived from conventional methods
*Brassica oleracea*	60	250	2	180	100 mL	765.9	sulforaphane and iberin nitrile	-	[72]	Dal Prá et al. investigated optimal conditions for extraction of antioxidant constituents of industrial interests
*Capcicum frutescens*	40	250	11.4	320	-	268.2	capsaicin	32.8 mg/g	[44]	Freeze-drying is the optimum sample pretreatment method to recover of capsaicinoids
*Cannabis sativa*	40–60	300–400	1.94 kg/h	-	-	-	tocopherol	125.37 μg/g	[37]	Extraction of α-tocopherol and γ-tocopherol
*Carica papaya*	80	200	16.45 mL/min	180	42 mL	1180.4	benzyl isothiocyanate	-	[49]	SFE is used to recover highly active compound from papaya seeds
*Camelina sativa*	70	450	1 L/min	510	-	16.14	α-linoleic, oleic, eicosaenoic and erusic acids	-	[54]	SFE method is more efficient in recovery of oil compared to hexane extraction and cold press
*Chenopodium Quinoa*	130	185	0.175–0.45	55–180	1.2 mL (0.5 cm × 6.1 cm)	8.02 to 67.5	tocopherol	201.3 mg/100 g	[73]	Four times increase in vitamin E yield compared with hexane extraction
*Chelidonium majus*	55	120	4.8	-	20 mL	-	chelidonine, cheleritrine, sanquinarine and berberine	-	[31]	Highly selective in extraction of chelidonine at solvent density of 813–850 kg/m^3^
*Coffea arabica*	35.9	331	70	-	1 L	-	palmitic, linoleic, oleic, strearic and arachidic acid, furans and pyrazine	-	[74]	Volatile compounds of furan and pyrazine type recovered from coffee beans
*Crocus sativus*	44.9	349	10.1 L/h	150	-	1377.27	-	-	[58]	Pressure and flowrate had significant effects on recovery of volatile compounds
*Dracocephalum kotschyi*	60	240	600	100	8.8 mm × 560 mm	22,058	citral, *p*-mentha-1,3,8-triene, D-3-carene and methyl geranate	-	[48]	CO_2_ and flowrate influence the extraction yield
*Expresso* Spent coffee ground	55	190	12	-	-	-	diterpenes	102.9 mg/g	[75]	Yield of diterpenes increased by 212–410%
*Eucalyptus globule*	80	350	12	60–120	200 mL	60.15 to 400	^†^ DPPH (antioxidant) and superoxide anion scavenging	26.05 and 47.61 μg/mL	[76]	Increase in temperature and flowrate augments the solubility and interaction of CO_2_ and essential oil
*Eucalyptus globulus*	40	200	6	360	-	1800	germacrenos D, germ-acrenos B + bicycle-germacrene, selina-1,3,7(11) -trien-8-one, selina-1,3,7(11)-trien-8-one epoxide, *trans*-caryophyllene	-	[77]	Study reported the recovery of triterpenic acids by 79.2% compared to soxhlet extraction
*Eugenia uniflora*	60	400	2.4	360	-	20.09	o-cymene, 1,8-cineole γ-terpinene, *cis*-sabinene (*trans*-4-thujanol), thymol-methyl ether, thymol carvacrol, α-copaene-*trans*-caryophyllene, germacrene D bicycle-germacrene, δ-cadinene, monoterpenes, sesquiterpenes	-	[78]	The authors showed that sequential extraction method is more effective in obtaining compounds of interest
*Euterpe oleracea (Residual pulp)*	FF	490	~5.4	180	-	21.41	linolenic, linoleic, oleic, and palmitic acids	-	[59]	The extracts obtained were more concentrated in monounsaturated fatty acid than polyunsaturated fatty acid
*Garcinia mangostana*	140–160	50–100	2 mL/min	30–60	8.8 mL	1.5–3	α-mangostin	-	[42]	SFE is used as means to extract α-mangostin with a yield of 0.203%
*Gynostemma pentaphyllum*	43	320	333.33	160	-	1483.11	linolenic acid	-	[55]	High content of unsaturated fatty acids is discovered (95.69%) compared with conventional methods
*Juniperus communis*	55	300	7	60	-	64.12	germacrene D and octadecene	-	[79]	Yield of seed oil is increased with sample particle diameter <0.315 mm
*Lippia sidoides* (sequential extraction)	60	400	0.5	360	-	4.19	-	-	[62]	Sequential extraction employing supercritical CO_2_ is effective in extraction of compounds of interest
*Maclura pomifera*	40	210	333.33	360	4 L	132.98	lupeol ester of 3-hydroxyhexadecanoic acid	-	[80]	Extraction of new compound, 3-hydroxyhexadecanoic acid
*Moringa oleifera*	60	500	2 mL/min	120	50 mL	37.85	1,2-benzenedicarboxylic acid, mono-(2-ethylhexyl) ester, nonacosane, heptacosane and β-amyrin	-	[81]	This work proved selectivity of SFE process by extracting 12 compounds compared to 42 compounds extracted by soxhlet extraction
*Moringa oleifera*	30	350	333.33	300	2 L	1329.77	oleic acid, tocopherols and sterol	-	[82]	A health promoting fatty acid; Oleic acid (72.26–74.72%) is extracted by SFE process
*Moringa oleifera* (microwave pretreatment)	40	300	166.7	210	1 L	921.23–1000.2	myristic acid, palmitic palmitoleic acid, stearic acid, oleic acid, linoleic acid, linolenic acid, arachidic acid, eicosenoic acid, behenic acid, lignoceric acid, saturated fatty acids, monounsaturated fatty acids, polyunsaturated fatty acids	-	[39]	Microwave irradiation technique is used as the sample pretreatment step for SFE and soxhlet extraction, and SFE extract is claimed to be of higher quality than the soxhlet extract
*Nigella damascena*	40	300	0.8	15	150 mL (ID = 30 mm)	0.8	germacrene A, damascenine and β-elemene	-	[83]	The yields of germacrene A and damascenine from SFE were 20% higher than with soxhlet extraction
*Silybum marianum (milk thistle)*	40	220	5 mL/min	150	150 mL (ID = 30 mm)	23.56	linoleic, oleic, palmitic acids, silychristin, silydianin, silibinin and taxifolin	-	[84]	The SFE extract showed potent cytotoxic effect against CaCo-2 cells
*Ocimum basilicum*	60	150	3.22	240	200 mL	91.89	linalool, eugenol, α-begamotene, germacrene D, γ-cadinene, δ-cardinene, β-selinene	-	[85]	This study used a drug exhaustion method to extract non-polar compounds
*Ocimum sanctum*	70	400	-	90	-	-	eugenol	0.463 g/100 g dry powder	[60]	SFE is used to obtain eugenol rich fraction as dry powder
*Paullinia cupana*	40	100		40	25 mL	-	-	20 mg/100 g	[86]	Ethanol is used as co-solvent to improve total phenolic content
*Persea Americana (Avocado)*	60	400		-	-	-	α-tocopherol	-	[61]	98% recovery of avocado oil from SFE extract was reported
*Phyllanthus amarus*	40	232		90	15 mm × 150 mm	-	phyllanthin	12.83 mg/g	[87]	Co-solvent concentration and extraction time significantly effect extraction yield
*Piper nigrum*	50	300	2 mL/min	80	50 mL	74.07	β-caryophyllene, limonene, cabinene, 3-carene and α-pinene	-	[88]	SFE extract exhibited a stronger radical scavenging activity compared with extract from hydrodistillation with EC50 of 103.28 and 316.27 µg /mL, respectively. Optimum parameter for antioxidant activity: T: 40 °C, time: 60 min
*Piper piscatorum*	40	400	3.6	30	-	35.18	piperovatine, palmitic acid, pentadecane, pipercallosidine	-	[89]	Chemical composition of extracts, particularly those containing amides was reduced when samples were air-dried
*Pleurotus ostreatus (Mushroom)*	48	210	333.33	90	100 mL	222,220	phenol content: 5.48 mg GAE/g (dry weight)	0.135 g dry weight	[90]	This study showed a good correlation between ergothioneine and ^†^ DPPH scavenging activity
*Rhodiola rosea*	62	317	0.4 mL/min	90	10 mL 60 mm × 15 mm	-	lotaustralin	2.05 g/kg	[91]	SFE is used to extract cynogenic glucoside compound “lotaustralin”
*Rosa canina (waste product)*	40	355	0.75 mL/min	90	10 mL	4090.91	palmitic acid, stearic acid, oleic acid, linoleic acid, arachidonic acid	0.0165 g dry solid	[92]	The highest extraction yield of oil was 16.5 g oil/100 g of dry solid
*Sasa palmate*	95	200	10 mL/min		9.65 mm × 45 mm	-	DL-alanine, gluconic acid, phosphoric acid, β-sitosterol, β-amyrene, α-amyrin acetate and friedelin	0.73 g catechin equivalent	[93]	The used of mixture of co-solvent in 1:3 ratio enhanced yield of polyphenols and radical scavenging activity
*Vitis vinefera (grape marc)*	40–60	251	167.7	180	-	2795	-	10.8 g/100 g	[94]	15% water as co-solvent efficiently extracts polar polyphenols from grape marc
*Wedelia calendulacea*	40	250		90	-	-	wedelolactone	0.008 g/100 g	[95]	SFE method is more selective to extract wedelolactone compared with Soxhlet extraction
*Zingiber officinale*	50	250	2 cm^3^/min	180	150 mL 25.2 mm × 290 mm	137.4	α-zingiberene, β-sesquiphellandrene, α-farnesene, geranial, β-bisabolene and β-eudesmol	2.62 g/100 g	[96]	SFE extract showed higher capacity in antimicrobial activity than hydrodistillation

Key: D = diameter (reactor), ID = internal diameter (reactor), L = length (reactor), * = based on % yield of g/100 g, FFA = free fatty acid, UFA = unsaturated fatty acids, ^†^ DPPH = 2,2-diphenyl-1-picrylhydrazyl, SFE = supercritical fluid extraction.

**Table 5:** Ultrasonic-Assisted supercritical fluid extraction.

Sample	SFE Conditions				Device/Sonication Power	Remark	Ref.
	Temp. (°C)	Pressure (bar)	Flow Rate (kg/s)	Time (min)			
*Syzygium aromaticum*	32	95	0.233 × 10^−4^	115	ultrasonic bath at 185 W	Yield of clove oil 11% higher by ultrasonic assisted SPE and 1.2 times increase in extraction of α-humulene	[110]
*Capsicum baccatum*	40	250	1.75 × 10^−4^	80	ultrasound probe at 600 W	Yield of capsai- cinoid increased up to 12%. Global yield increased up to 45%	[111]
*Capsicum frutescens*	40	150	1.673 × 10^−4^	60	ultrasound probe at 360 W	Global yield increased up to 77%	[112]
*Hedyotis diffusa*	55	245	-	95	ultrasonic bath at 185 W	Yield increased by 11–14%	[113]
*Perilla frutescens*	25	100	-	60	ultrasound irradiation 125 s	Yield of luteolin increased by 53% Yield of apigenin increased by 144%	[41]
Almond Oil (source unknown)	55	280	55.6 × 10^−4^	510	ultrasonic probe	Extraction yield of the oil was enhanced by 20%	[108]
*Zingiber Officinale*	40	160	-	200	unknown device at 300 W	Yield: 30% higher	[114]
